# The Role of Aquaporin 7 in the Movement of Water and Cryoprotectants in Bovine In Vitro Matured Oocytes

**DOI:** 10.3390/ani12040530

**Published:** 2022-02-21

**Authors:** Tania García-Martínez, Iris Martínez-Rodero, Joan Roncero-Carol, Meritxell Vendrell-Flotats, Jaume Gardela, Alfonso Gutiérrez-Adán, Priscila Ramos-Ibeas, Adam Z. Higgins, Teresa Mogas

**Affiliations:** 1 Department of Animal Medicine and Surgery, Autonomous University of Barcelona, ES-08193 Cerdanyola del Vallès, Spain; taniagarciamartinez@gmail.com (T.G.-M.); iris.martinez@outlook.com (I.M.-R.); joanroncerocarol@gmail.com (J.R.-C.); meritxell.vflotats@gmail.com (M.V.-F.); 2 Department of Animal Health and Anatomy, Autonomous University of Barcelona, ES-08193 Cerdanyola del Vallès, Spain; jaume.gardela@uab.cat; 3 Department of Animal Reproduction, National Institute for Agriculture and Food Research and Technology (INIA), Avda. Puerta de Hierro 12, Local 10, 28040 Madrid, Spain; agutierr@inia.es (A.G.-A.); ramos.priscila@inia.es (P.R.-I.); 4 School of Chemical, Biological and Environmental Engineering, Oregon State University, Corvallis, OR 97331-2702, USA; adam.higgins@oregonstate.edu

**Keywords:** cell membrane permeability, cryoprotective agents, artificial protein expression, aquaporin 3, aquaporin 9, cryopreservation, dimethyl sulfoxide, ethylene glycol, facilitated diffusion

## Abstract

**Simple Summary:**

The permeability of the plasma membrane to water and cryoprotectants is a critical factor in the effective vitrification of oocytes. The goal of this study is to better understand the pathways used to transport water and other cryoprotectants through the plasma membrane of bovine in vitro matured oocytes, with a focus on the role of aquaporin 7 (AQP7). We demonstrated that cryoprotectants stimulated AQP3 and AQP7 but not AQP9 expression in mature bovine oocytes. Dimethyl sulfoxide upregulates AQP3 expression, while ethylene glycol upregulates AQP7 expression in oocytes in a CPA-dependent fashion. We also demonstrated that exogenous expression of aquaglyceroporins such as AQP7 is possible in in vitro matured oocytes. When permeability values for membrane transport of dimethyl sulfoxide, ethylene glycol and sucrose were assessed, we observed that AQP7 overexpressed oocytes are more permeable to water in the presence of dimethyl sulfoxide solution. These biophysical characteristics, together with the use of membrane transport modeling, will allow re-evaluation and possibly improvement of previously described protocols for bovine oocyte cryopreservation.

**Abstract:**

Aquaglyceroporins are known as channel proteins, and are able to transport water and small neutral solutes. In this study, we evaluate the effect of exposure of in vitro matured bovine oocytes to hyperosmotic solutions containing ethylene glycol (EG), dimethyl sulfoxide (Me_2_SO) or sucrose on the expression levels of AQP3, AQP7 and AQP9. Moreover, we studied whether artificial protein expression of AQP7 in bovine oocytes increases their permeability to water and cryoprotectants. Exposure to hyperosmotic solutions stimulated AQP3 and AQP7 but not AQP9 expression. Oocytes exposed to hyperosmotic Me_2_SO solution exhibited upregulated AQP3 expression, while AQP7 expression was upregulated by EG hyperosmotic exposure. Microinjection of oocytes at the germinal vesicle stage with enhanced green fluorescent protein (EGFP) or EGFP+AQP7 cRNAs resulted in the expression of the corresponding proteins in ≈86% of the metaphase-II stage oocytes. AQP7 facilitated water diffusion when bovine MII oocytes were in presence of Me_2_SO solution but not EG or sucrose solution. However, the overexpression of this aquaporin did not increase membrane permeability to Me_2_SO or EG. In summary, cryoprotectant-induced increase of AQP3 and AQP7 expression could be one of the mechanisms underlying oocyte tolerance to hyperosmotic stress. Water diffusion appears to be improved when AQP7 overexpressed oocytes are exposed to Me_2_SO, shortening the time required for oocytes to achieve osmotic balance with cryoprotectant solutions.

## 1. Introduction

Recent developments in vitrification technology have enhanced the efficiency of bovine oocyte cryopreservation in terms of oocyte survival and embryo development rates after warming. However, there is still potential for improvement in terms of vitrification methods [1,2]. One of the key issues in cryobiology is the potentially lethal mechanical stresses that cells experience when exposed to high-osmolarity solutions that enable exchange of intracellular water with cryoprotectant agents (CPAs). This concern about such osmotic stress is especially acute for the ultra-fast-cooling technique known as vitrification, which uses high concentrations of CPAs to prevent the formation of lethal intracellular ice crystals [3]. When compared to other mammalian cells, oocytes with diameters on the order of 100 µm have ~1000 times larger volume of water that needs to be replaced with CPAs. However, the area of the cell surface does not increase correspondingly, which results in slower water–CPA exchange in oocytes and makes the cell very susceptible to osmotic stress [4,5]. Therefore, the movement of water and solutes across the cell membrane plays a crucial role in cell viability because it influences the major forms of cell injury caused by cryopreservation, including damage from intracellular ice crystal formation, CPA toxicity, and osmotic stress during the addition and removal of the CPA [6,7].

Two different pathways facilitate the movement of water and CPAs across the plasma membrane, simple diffusion through the lipid bilayer, and facilitated diffusion through channels [8]. Movement through simple diffusion is characterized by low permeability to water (hydraulic conductivity [*L*_p_]) or to a CPA (*P_s_*), but permeability highly depends on the temperature. Therefore, longer exposure to the cryopreservation solution is necessary to allow the cell to dehydrate and facilitate CPA diffusion, which increases the potential for CPA toxicity [9,10]. Conversely, the permeability would be higher when water and CPAs permeate the plasma membrane by facilitated diffusion through channels. Facilitated diffusion is also less affected by temperature. However, in both cases, exposure time to the cryopreservation solution needs to be limited to avoid damage from the chemical toxicity of the CPA [11]. Therefore, rapid movement of water and CPAs through the plasma membranes is essential to minimize damaging exposure times to CPAs.

Aquaporins, present in all living organisms, from bacteria to mammals, are a superfamily of small (25–34 kDa), hydrophobic, integral membrane proteins that facilitate rapid, passive movement of water across cellular membranes [12]. To date, thirteen isoforms of aquaporins (AQP0–12) have been identified, and while most of them are water-selective, functional studies have identified a subgroup of channels, termed as aquaglyceroporins (AQP3, AQP7, AQP9 and AQP10), that are non-selective water channels, and can transport glycerol, urea and other small non-electrolytes as well as water [13,14]. The expression and function of AQPs in mammalian oocytes are poorly understood. Few studies have focused on the expression of AQPs in oocytes from different species and the role of AQPs (besides the probable effect on water and ionic regulation) remains to be elucidated. Edashige et al. were pioneers in this field. According to their findings in mouse and bovine oocytes, water and CPAs predominantly move through oolemma by simple diffusion, with a lower proportion of water moving through channel processes; hence, AQP3 has been described as playing a significant role in helping the diffusion of water, glycerol, and ethylene glycol (EG) in cow and mouse oocytes [15,16]. Exogenous expression of rat AQP3 in mouse oocytes or human and zebrafish AQP3 in porcine oocytes was also tested to see if artificial expression of AQP3 could increase water and CPA permeability and oocyte survival after cryopreservation [17,18,19]. In these studies, exogenous expression of AQP3 improved oocyte cryotolerance and survival and embryo development after warming of mouse oocytes by increasing both the outflow of water and the influx of CPAs into the cell [18,19]. However, there is no information in the literature regarding the expression of other aquaglyceroporins and their effect on the permeability to water and CPAs in bovine oocytes.

The aim of this study was to examine the pathways for the movement of water and cryoprotectants through bovine in vitro matured oocytes with special reference to the role of AQP7. We first investigated the expression of AQP3, AQP7 and AQP9 after exposure of in vitro matured bovine oocytes to hyperosmotic CPA solutions. Then we examined the role of AQP7 in the movement of water and cryoprotectants in bovine in vitro matured oocytes in which AQP7 was artificially overexpressed through cRNA injection.

## 2. Materials and Methods

### 2.1. Chemicals

Unless indicated otherwise, all chemicals were purchased from Sigma Chemical Co. (St. Louis, MO, USA).

### 2.2. Oocyte Collection and In Vitro Maturation

The methodology used for the in vitro maturation (IVM) of the bovine oocytes has been described elsewhere [20]. Cow ovaries were collected from a local slaughterhouse, placed in a thermos containing saline (0.9% *w*/*v* NaCl), and transferred to the laboratory at 35–37 °C within 1 h. The cumulus–oocyte complexes (COCs) were collected by aspiration of 3- to 8-mm follicles. Only those COCs with a compact cumulus and homogeneous dark ooplasm were selected for in vitro maturation (IVM). After adding 500 μL of maturation medium to each well in a 4-well dish (Nunc, Roskilde, Denmark), groups of up to 50 COCs were transferred to each well and cultured for 24 h. The composition of the maturation medium (IVM medium) was as follows: 10% (*v*/*v*) fetal bovine serum (FBS), 10 ng/mL epidermal growth factor and 50 μg/mL gentamicin added to tissue culture medium (TCM-199). All IVM procedures were performed in a 38.5 °C humid incubator with 5% CO_2_.

### 2.3. Analysis of AQP3, AQP7 and AQP9 Expression

After 24 h of IVM, bovine oocytes were completely denuded of cumulus cells by gentle pipetting and exposed to holding medium (HM: TCM199-Hepes supplemented with 20% (*v*/*v*) FBS) containing 9.5% (*v*/*v* 1.2 M) Me_2_SO, 8% (*v*/*v* 1.3 M) EG, or 0.5 M sucrose at 25 °C during 20 min [21,22]. Oocytes kept in HM served as controls. Oocytes were then denuded of cumulus cells by gentle pipetting and fixed in 2% (*w*/*v*) paraformaldehyde–phosphate buffer saline (PFA-PBS) for 30 min; all steps were performed at 38.5 °C unless otherwise stated. After washing in PBS, oocytes were permeabilized in Triton X-100 (2.5% (*v*/*v*) in PBS) for 20 min and blocked in 3% bovine serum albumin (BSA) (*w*/*v*) in PBS for 30 min. After washing, oocytes were incubated either with rabbit anti-AQP3 antibody (70R-51452, Fitzgerald, USA) (1:200), rabbit anti-AQP7 antibody (NBP1–30862; Novus Biologicals, USA) (1:200) or rabbit anti-AQP9 antibody (GTX37783, GeneTex, USA) (1:200) overnight at 4 °C, followed by incubation with goat Alexa Fluor™ 488-conjugated anti-rabbit IgG antibody (A11034, Life technologies, Carlsbad, CA, USA) (1:700) for 1 h. Following secondary antibody incubation, oocytes were washed x3 in PBS supplemented with 0.005% (*v*/*v*) Triton X-100 for 20 min. As negative control, primary antibodies were omitted. Nuclei were stained with 125 ng/mL 4′,6′-diamidino-2-phenylindole hydrochloride (DAPI) (Vysis Inc., Downers Grove, IL, USA) in Vectashield mounting medium. A laser-scanning confocal microscope (Leica TCS SP5, Leica Microsystems, Mannheim, Germany) was used to examine AQP3, AQP7 or AQP9 (Alexa Fluor™ 488; excitation 488 nm), and chromatin (DAPI; excitation 405 nm) staining.

Fluorescence intensities of the oocytes were analyzed after subtraction of the background fluorescence using ImageJ software (Version 2.0.0-rc-69/1.52p; National Institutes of Health, Bethesda, MD, USA) and normalized to those of the control oocytes. A total of 25–40 oocytes (in three different replicates) of one representative experiment were analyzed.

### 2.4. Preparation of AQP7 cRNA

**Figure 1:**
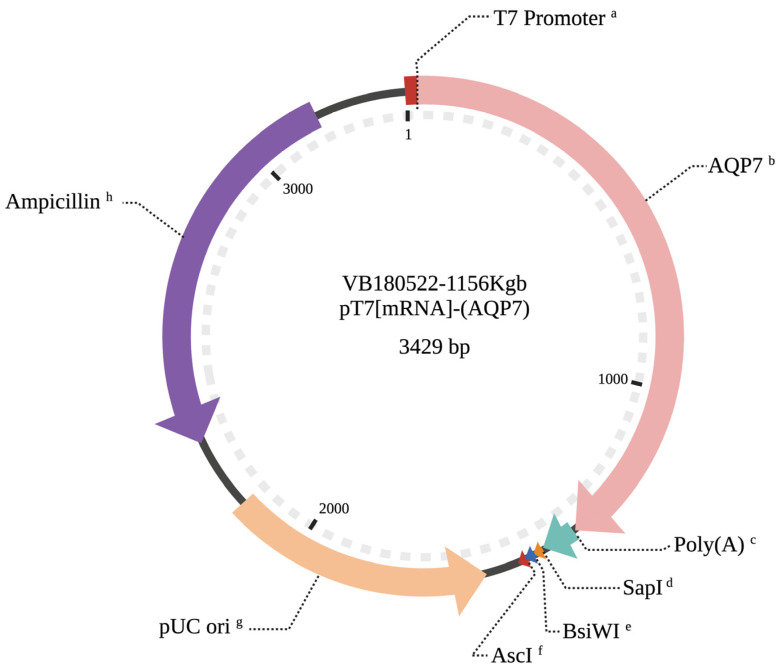
Composition of the pT7/bovine AQP7 plasmid. ^a^ T7 RNA polymerase promoter. ^b^ AQP7 transcribed sequence. ^c^ Synthetic poly(A)30 region. ^d^ SapI restriction site. ^e^ BsiWI restriction site. ^f^ AscI restriction site. ^g^ pUC origin of replication. ^h^ Ampicillin resistance gene.

A circular plasmid constructed and packaged by VectorBuilder (Santa Clara, CA, USA) was used to drive the AQP7 expression in metaphase II (MII) oocytes following cytoplasmic microinjection at the germinal vesicle (GV) stage. The AQP7 vector (id: VB180522-1156Kgb) (Figure 1) contained the active AQP7 gene sequence (GenBank accession number NM_001076378.2). The AQP7 gene sequence was under the control of the T7 promoter. The vector had a PolyA signal (SV30) after the AQP7 sequence, which facilitates transcription termination and polyadenylation of mRNA transcribed by PolII RNA polymerase. In addition, the circular vector contained a 861 bp region including the ampicillin resistance gene. The green fluorescent protein (EGFP) expression vector pcDNA3.2EGFP-poly(A83) [23] was used as reporter gene.

AQP7 expression vector was digested by AscI (New England Biolabs, Ipswich, MA, USA) and EGFP expression vector pcDNA3.2EGFP-poly(A83) was digested by XhoI and XbaI (New England Biolabs, Ipswich, MA, USA). Then, AQP7 and EGFP cRNAs were in vitro transcribed with mMESSAGE mMACHINE T7 Ultra Kit (Life technologies, Carlsbad, CA, USA) [24] and purified using FavorPrep^TM^ Tissue Total RNA Mini Kit (Favorgen, Viena, Austria). The cRNAs were prepared at 50 ng/μL (AQP7 cRNA) and 30 ng/μL (EGFP cRNA) in water.

### 2.5. Microinjection of AQP7 cRNA into Bovine Oocytes

Immature bovine oocytes at the GV stage were collected from ovaries bathed in PBS. In each experiment, 150 oocytes were pooled and used. Oocytes were denuded of cumulus cells by repeatedly pipetting them in PBS medium. Groups of 20 denuded oocytes with normal size (>115 and <130 microns) and homogeneous dark color were placed in 25 μL drops of HM covered with mineral oil in a Petri dish (60 × 15 mm). One oocyte was immobilized with a holding pipette (outer diameter, 100 µm; MPHL-35, Life Global group, Guiford, NC, USA) connected to a micromanipulator on an inverted microscope (Zeiss Axio Vert A1, Germany) and injected with an injection needle (MPIC-30L, Life Global group, Guiford) connected to another micromanipulator. Oocytes were injected with ~10 pL of water solution containing EGFP cRNA alone (30 ng/μL), or together with AQP7 cRNA (50 ng/μL). Non-injected oocytes (intact oocytes) served as control. Both non-injected and injected oocytes were cultured at 38.5 °C in a humidified CO_2_ incubator (5% CO_2_) for up 24 h in IVM medium. Microinjected oocytes were supplemented with intact COCs (1:1 ratio) during in vitro maturation to improve the developmental capability of oocytes deprived of their CCs investment [25]. Oocyte survival was assessed after IVM and only oocytes showing a polar body were classified as matured and used as non-injected (control), EGFP cRNA-injected or EGFP+AQP7 cRNA-injected oocytes. Oocyte survival was evaluated on the basis of the integrity of the oocyte membrane and the zona pellucida together with the discoloration of the cytoplasm.

### 2.6. Expression of AQP7 in cRNA-Injected Oocytes

At 24 h of IVM, cumulus-free non-injected (control), EGFP cRNA-injected or EGFP+AQP7 cRNA-injected oocytes showing the first polar body were fixed in 2% (*w*/*v*) PFA-PBS for 30 min. Unless otherwise noted, all processes were completed at 38.5 °C. After washing in PBS, oocytes were permeabilized for 20 min in Triton X-100 (2.5% (*v*/*v*) in PBS) and blocked for 30 min in 3% BSA (*w*/*v*) in PBS. After washing, oocytes were incubated with rabbit anti-AQP7 antibody (NBP1–30862; Novus Biologicals, USA) (1:200) overnight at 4 °C, followed by incubation with goat Alexa Fluor™ 568-conjugated anti-rabbit IgG antibody (A11011, Life technologies, Carlsbad, CA, USA) (1:700) for 1 h. Oocytes were then washed x3 in PBS supplemented with 0.005% (*v*/*v*) Triton X-100 for 20 min. As negative control, the primary antibody was omitted. Nuclei were stained with 125 ng/mL DAPI in Vectashield mounting medium. A laser-scanning confocal microscope (Leica TCS SP5, Leica Microsystems, Mannheim, Germany) was used to examine AQP7 (Alexa Fluor™ 568; excitation 568 nm) and chromatin (DAPI; excitation 405 nm) staining. AQP7 and nuclei were red and blue, respectively, in the overlay image created by combining the different channels. When EGFP was present, it was detected using a 488-nm excitation and the ooplasm was dyed green. Fluorescence intensities of the oocytes were analyzed after deduction of the background fluorescence using ImageJ software and normalized to those of the control oocytes. A total of 20–30 oocytes (in three different replicates) were analyzed for each experimental group.

### 2.7. Measurement of Permeability to Water and Cryoprotectants

After 22–24 h of culture, non-injected (control), EGFP cRNA-injected or EGFP+AQP7 cRNA-injected oocytes with the first polar body were striped of cumulus cells by gentle pipetting and used for permeability studies following the methods published previously for mouse oocytes [19] with slight changes.

In brief, each oocyte was suspended in a 25 μL drop of HM covered with mineral oil, and was maintained in place by a holding pipette (outer diameter, 95–120 µm; MPH-MED-30, Origio, Denmark) attached to a micromanipulator on an inverted microscope (Zeiss Axio Vert A1, Germany). To calculate the initial volume, an initial photograph of the oocyte was taken. The oocyte was subsequently covered with a pipette with a bigger inner diameter (600-µm diameter) (G-1 Narishigue, Tokyo, Japan) attached to a separate micromanipulator. Then, by sliding the dish and removing the pipette that was covering it, the oocyte was abruptly exposed to 25 µL drop of HM containing 0.5 M sucrose, 9.5% (*v*/*v* 1.2 M) Me_2_SO or 8% (*v*/*v* 1.3 M) EG at 25 °C during 5 min. Permeability values of non-injected and cRNA-injected oocytes to water and CPAs were determined from changes in oocyte volume while suspended in HM containing sucrose, Me_2_SO or EG for 5 min at 25 °C. A time-lapse video recorder (Zeiss Zen imaging software/Axiocam ERc 5s) was used to record the oocyte’s cell volume response every 5 s. The volume of the oocyte in each image was calculated from the area of the cross section using ImageJ software (National Institutes of Health, Bethesda, MD, USA). Only oocytes that maintain an approximate spherical geometry were individually analyzed. The *L*_p_ and *P*_s_ of the bovine oocyte were estimated, based on the change in concentration of the solutes and the original volume of the oocyte, by fitting the movement of water and cryoprotectants by using a the previously described two-parameter (2P) formalism [26,27,28].

Briefly, the 2P model describes osmotic responses of cells in fluids containing both permeable and nonpermeable solutes. The water flux into the cell over time is expressed in this formalism as:

(1) $$\frac{dV_{w}}{dt}=-L_{p}ART\left(M^{e}-M^{i}\right)$$

where *V_w_* is the cell water volume, *L_p_* is the membrane hydraulic conductivity, *A* is the area of the plasma membrane, *R* is the universal gas constant, *T* is the absolute temperature, and *M^e^* and *M^i^* are the total external and internal osmolalities, respectively.

The rate of CPA transport is given by:

(2) $$\frac{dN_{s}}{dt}=P_{s}A\left(M_{s}^{e}-M_{s}^{i}\right)$$

where *N_s_* is the intracellular moles of CPA, *P_s_* is the CPA permeability, $M_{s}^{i}$ and $M_{s}^{e}\;$ are the intracellular and extracellular CPA molality, respectively. It is necessary to multiply by the partial molar volume of the CPA, *υ_s_*, to obtain the intracellular CPA volume, resulting in

(3) $$V_{s}=\upsilon_{s}N_{s}$$

Then, the total cell volume (*V_c_*) is just the sum of the water (*V_w_*), CPA (*V_s_*), and solids (*V_b_*) volumes:

(4) $$V_{c}=V_{w}+V_{s}+V_{b}$$

The ode45 function, which implements an explicit Runge-Kutta formula, was used to solve the differential equations (Equations (1) and (2) for the 2P model) in MATLAB software [29,30]. To estimate the permeability values, model predictions were fit to the data by minimizing the sum of the error squared in MATLAB using the fminsearch function, which implements the Nelder-Mead simplex algorithm [31]. Table 1 lists the various constants and parameters that appear in the equations.

### 2.8. Experimental Design

Experiment 1. Analysis of AQP3, AQP7 and AQP9 expression in oocytes after treatment with cryoprotection solutions.

To analyze AQP3, AQP7 and AQP9 expression after exposure of in vitro bovine oocytes to hyperosmotic CPA solutions, IVM bovine oocytes were treated with holding medium containing 9.5% (*v*/*v*) Me_2_SO, 8% (*v*/*v*) EG, or 0.5 M sucrose for 20 min at 25 °C. Oocytes kept in holding medium were used as controls. The oocytes were then collected and fixed immediately. The relative immunofluorescence levels of AQP3, AQP7 or AQP9 were analyzed by immunofluorescence.

Experiment 2. Measurement of permeability to water and cryoprotectants of in vitro matured oocytes in which AQP7 was artificially overexpressed.

To determine whether bovine oocytes could translate AQP7, GV-stage oocytes were injected with EGFP or EGFP+AQP7 cRNA; then, immunofluorescence was used to measure AQP7 protein expression after in vitro oocyte maturation. As an additional control, a third batch of non-injected oocytes was used.

For permeability measurements, cumulus-free IVM oocytes from control (non-injected oocytes), EGFP, and EGFP+AQP7 groups were freed of cumulus cells by gentle pipetting and abruptly exposed to HM containing 0.5 M sucrose, 9.5% (*v*/*v*) Me_2_SO or 8% (*v*/*v*) EG at 25 °C during 5 min. The cell volume response of the oocyte during the experiments was recorded every 5 s with a time-lapse video recorder. The volume of the oocyte in each image was calculated from the area of the cross section using ImageJ software.

### 2.9. Statistical Analysis

All data sets were analyzed for normalcy and homoscedasticity using the Shapiro–Wilk normality test and Levene’s test, respectively. Non-normal data distribution was restored using log(x + 1) transformation prior to analysis. To determine if there was a significant relationship between the maturation data and the groups of the study, data were analyzed by Fisher’s exact test. Additionally, the relationship between the % survival and % EGFP in the microinjected groups was also analyzed. The immunofluorescent and permeability data were analyzed by one-way ANOVA tests, followed by Tukey’s multiple comparison test. A non-parametric alternative (Kruskal–Wallis test) was used in data that did not meet the assumptions of the one-way ANOVA test, followed by pairwise Wilcoxon rank-sum tests. R software version 3.6.1 [34] was used to conduct the statistical analyses. The threshold for significance was set at *p* < 0.05.

## 3. Results

### 3.1. Analysis of AQP3, AQP7 and AQP9 Expression in Oocytes after Treatment with Cryoprotection Solutions

**Figure 2:**
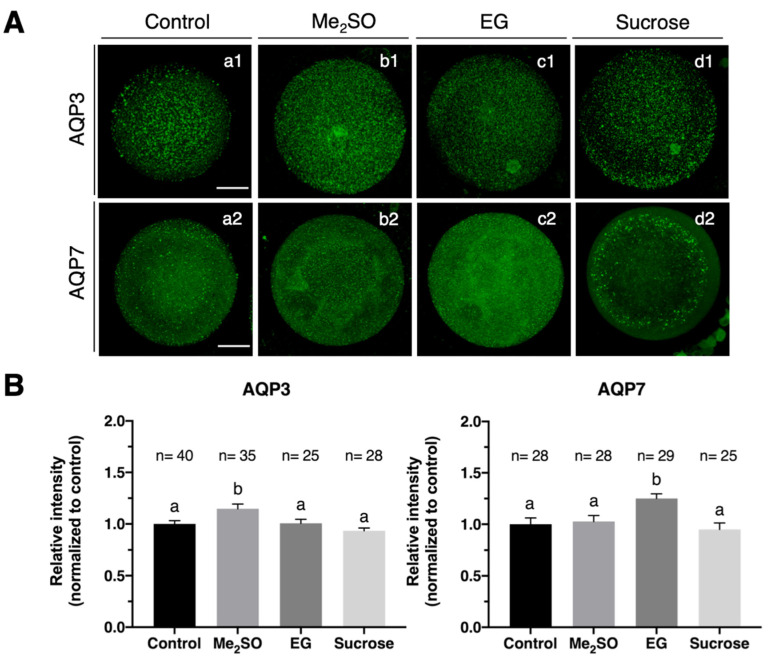
Effect of the exposure of IVM bovine oocytes to hyperosmotic CPA solutions on the levels of expression of AQP3 and AQP7. (**A**) Immunofluorescence analysis of AQP3 and AQP7 expression in bovine oocytes without treatment (a1 and a2, respectively) and in the presence of 9.5% Me_2_SO (b1 and b2, respectively), 8% EG (c1 and c2, respectively) or 0.5 M sucrose (d1 and d2, respectively). The concentrations of the two penetrating cryoprotectants, EG and Me_2_SO, were chosen to prepare solutions with similar osmolalities, as previously indicated [15]. AQP3 and AQP7 protein expression is displayed in green. Scale bar, 30 μm. (**B**) Summary data of the relative immunofluorescence intensity normalized to control. Data are presented in arbitrary units ± SEM, *n* ≥ 25, ^a,b^ Different letters indicate statistically significant differences between treatments (*p* < 0.05).

We treated bovine oocytes with a high concentration of two penetrating cryoprotectants, EG and Me_2_SO, and a non-penetrating cryoprotectant, sucrose, to examine if hyperosmotic stress induced changes in aquaglyceroporin expression. Immunofluorescence revealed that AQP7 protein expression increased (*p* *<* 0.05) when bovine oocytes were exposed to EG while no changes in AQP7 fluorescence intensity was observed when oocytes were exposed to Me_2_SO or sucrose when compared to the control group. Additionally, exposure to Me_2_SO significantly (*p* *<* 0.05) increased the relative fluorescence intensity of AQP3 protein expression, while no changes were observed after exposure to EG or sucrose (Figure 2B). Quantification of relative fluorescence intensity after exposure of IVM bovine oocytes to EG, Me_2_SO or sucrose showed no significant effect on AQ9 localization or abundance compared to the control.

### 3.2. Measurement of Permeability to Water and Cryoprotectants of In Vitro Matured Oocytes in Which AQP7 Was Artificially Overexpressed

#### 3.2.1. Expression of AQP7 in cRNA-Injected Oocytes

The results shown in Figure 2 suggest that hyperosmotic stress may upregulate AQP3 and AQP7 expression in bovine oocytes selectively, and both aquaglyceroporins may play a role in water and cryoprotectant transport during oocyte cryopreservation. Although various studies on the AQPs expression and function in bovine oocytes have been carried out, the majority of studies focus on the expression and role of AQP3 in the movement of water and cryoprotectants [16,19]. To elucidate whether AQP7 is involved in the movement of water and CPA in bovine in vitro matured oocytes, we artificially over-expressed AQP7 by injecting them with AQP7 cRNA at the GV-stage.

**Figure 3:**
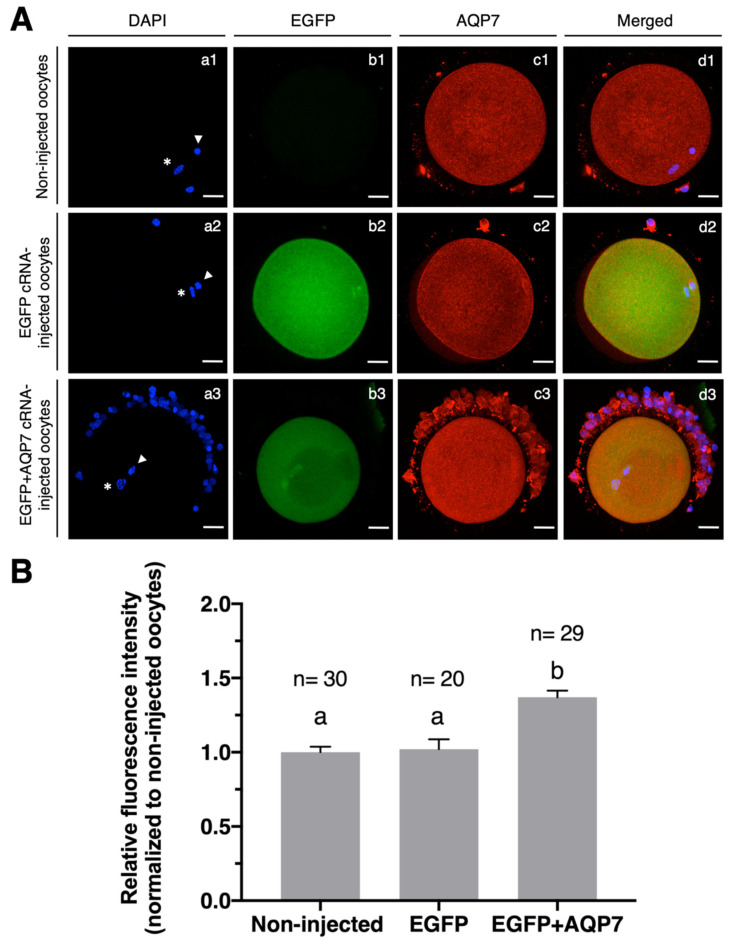
Exogenous expression of EGFP and EGFP+AQP7 cRNA in bovine MII oocytes. (**A**) Immunofluorescence analysis of AQP7 expression in bovine oocytes of non-injected, EGFP cRNA-injected and EGFP+AQP7 cRNA-injected oocytes. Nuclei counterstained with DAPI are displayed in blue (a1–a3), EGFP expression is displayed in green (b1–b3) and AQP7 protein expression is displayed in red (c1–c3). Overlayed images are shown in (d1–d3). White asterisk indicates chromosomes; white arrowhead indicates polar body. Scale bar, 30 μm. (**B**) Summary data of the relative immunofluorescence intensity normalized to control. Data are presented in arbitrary units ± SEM, *n* ≥ 20, ^a,b^ Different letters indicate statistically significant differences between treatments (*p* < 0.05).

The relative fluorescence intensity of AQP7 protein was significantly higher in oocytes injected with EGFP+AQP7 cRNA (1.37 ± 0.04) when compared to non-injected or EGFP cRNA-injected oocytes (1.02 ± 0.07), while no difference in relative immunofluorescence intensity was observed for AQP7 in EGFP cRNA-injected and non-injected oocytes (1.02 ± 0.07 and 1 ± 0.04, respectively) (Figure 3). These results indicate that AQP7 protein can be synthesized and overexpressed in bovine oocytes during in vitro maturation.

Co-injection of EGFP cRNA with cRNA encoding AQP7 and further immunostaining for AQP7 demonstrated that 100% of oocytes expressing EGFP also co-expressed AQP7 (Table 2). Immunofluorescence staining of AQP7 showed highly specific expression in the ooplasm and around the oolemma of EGFP+AQP7 cRNA-injected oocytes. Although we were able to determine its expression near the oolemma, we could not clearly confirm AQP7 expression in the oolemma. However, oocytes overexpressing AQP7 exhibited almost two-fold higher water permeability after exposure to Me_2_SO, which suggests that AQP7 protein is located in the oolemma.

The percentage of oocytes that survived the microinjection process was 71.48 ± 8.15% and 62.11 ± 2.25% for EGFP and EGFP+AQP7 cRNA-injected oocytes, respectively (Table 2). When EGFP protein expression was assessed in live oocytes after IVM, no differences between EGFP and EGFP-AQP7 cRNA-injected oocytes were observed (88.31 ± 9.36% and 82.88 ± 4.25%, respectively). The maturation rates of both EGFP and EGFP+AQP7 cRNA-injected oocytes after 24 h of culture, measured as the number of oocytes that reached the metaphase II stage over the total number of survived oocytes, was significantly reduced by approximately 20% with respect to the noninjected oocytes (Table 2).

Due to the similar expression efficiency of AQP7 and EGFP in bovine oocytes, only those AQP7 cRNA-injected oocytes that co-expressed EGFP were chosen for the permeability studies [35].

#### 3.2.2. Permeability to Water and Cryoprotectants of AQP7 cRNA-Injected Oocytes

**Figure 4:**
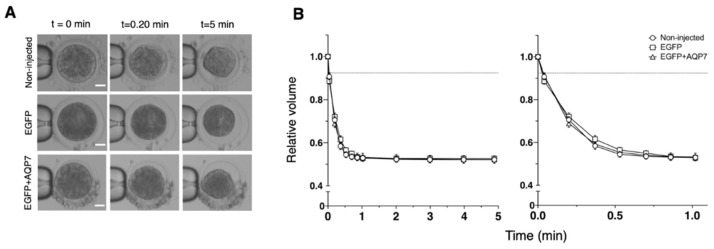
Osmotic behavior of non-injected MII bovine oocytes (circles), EGFP cRNA-injected (squares), or EGFP+AQP7 cRNA-injected-oocytes (triangles) exposed to 0.5 M sucrose for 5 min at 25 °C. (**A**) Representative phase-contrast microscopy images of oocytes measured in B. Scale bar, 30 μm. (**B**) Summary data represented as the relative mean volume ± SEM. Non-injected oocytes: *n* = 10; EGFP cRNA-injected oocytes: *n* = 12 and EGFP+AQP7 cRNA-injected oocytes: *n* = 12.

We measured changes in the cell volume of oocytes during 5 min of exposure to HM containing 0.5 M sucrose at 25 °C to determine if expression of EGFP alone or in combination with AQP7 increased the water permeability properties of bovine MII oocytes (Figure 4). Overall, in a hypertonic sucrose solution, oocytes from all groups shrank quickly during the first 30 s and then slowly approached an equilibrium volume of about 55% of the isotonic volume (Figure 4B). Consequently, the hydraulic conductivity (*L*_p_) calculated from the volume changes of the treated oocytes was similar to that of the non-injected oocytes (Table 3).

**Figure 5:**
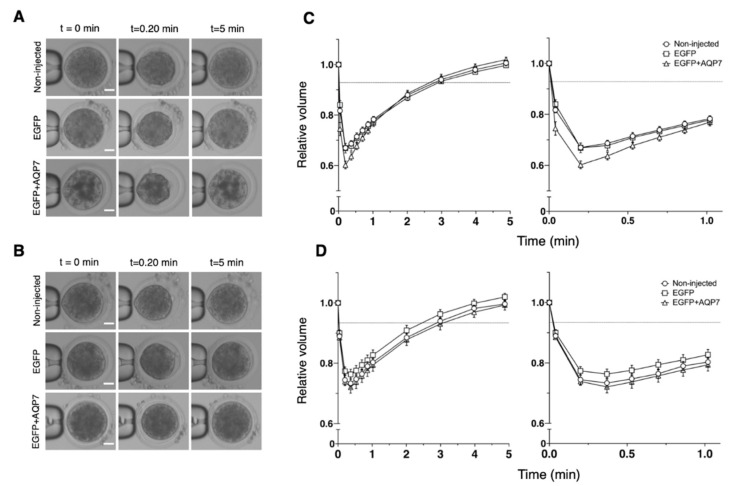
Osmotic behavior of non-injected MII bovine oocytes (circles), EGFP cRNA-injected (squares), or EGFP+AQP7 cRNA-injected-oocytes (triangles) exposed to 9.5% Me_2_SO or 8% EG for 5 min at 25 °C ((**A**,**B**), respectively). (**A**,**B**) Representative phase-contrast microscopy images of oocytes measured in C and D are shown. Scale bar = 30 μm. (**C**,**D**) Summary data represented as the relative mean volume ± SEM. Non-injected oocytes: Me_2_SO *n* = 13; EG *n* = 14; EGFP cRNA-injected oocytes: Me_2_SO *n* = 10; EG *n* = 11; and EGFP+AQP7 cRNA-injected oocytes: Me_2_SO *n* = 11; EG *n* = 13.

To evaluate if the exogenous expression of AQP7 in oocytes can also improve cryoprotectant permeability, changes in the cell volume of non-injected, EGFP cRNA or EGFP+AQP7cRNA-injected bovine oocytes during 5 min of suspension in HM containing 9.5% Me_2_SO or 8% EG at 25 °C were assessed (Figure 5). In a dimethyl sulfoxide solution, EGFP+AQP7 cRNA-injected oocytes shrank quite rapidly to 60% of their isotonic volume in 12 s, but regained their volume slowly (reaching 95% of the isotonic volume in 3 min). On the other hand, non-injected and EGFP cRNA-injected oocytes exhibited slightly slower shrinkage after exposure to Me_2_SO solution (Figure 5B). In accordance with the changes in oocyte volume, the *P*_Me2SO_ values for non-injected, EGFP cRNA and EGFP+AQP7 cRNA-injected oocytes were similar (0.58 ± 0.05, 0.58 ± 0.05 and 0.57 ± 0.04 μm/s, respectively) (Table 5). Nevertheless, the *L*_p_ value for EGFP+AQP7 cRNA-injected oocytes (3.96 ± 0.57 μm/atm×min) was much higher than that of non-injected (2.06 ± 0.13 μm/atm×min) and EGFP cRNA-injected (1.98 ± 0.24 μm/atm×min) oocytes (Table 4).

In an ethylene glycol solution, oocytes shrank relatively slowly (reaching 72–76% of the isotonic volume in 22 s) and regained their volume slowly (reaching 93–96% of the isotonic volume in 3 min), regardless of the injection of EGFP or EGFP+AQP7 cRNA (Figure 5D). Therefore, the *L*_p_ and *P*_EG_ values for EGFP+AQP7 cRNA-injected oocytes (1.39 ± 0.11 μm/atm×min and 0.76 ± 0.10 μm/s, respectively) was similar to that for non-injected (1.38 ± 0.12 μm/atm×min and 0.78 ± 0.09 μm/s, respectively) and EGFP cRNA-injected (1.44 ± 0.10 μm/atm×min and 1.04 ± 0.17 μm/s, respectively) oocytes (Table 4 and Table 5).

## 4. Discussion

The plasma membrane’s permeability to water and CPAs is critical for cell survival during cryopreservation. Water and CPA transport rates across the plasma membrane are low in simple diffusion, but they are significantly higher in facilitated diffusion where AQPs are involved [16]. Because aquaglyceroporins are permeable not only to water, but also to small neutral solutes, AQPs, and especially members of the aquaglyceroporin subfamily, which includes AQP3, AQP7 and AQP9, are essential for preventing osmotic-induced damage [14,36,37]. In the present study, we demonstrated that cryoprotectants stimulated AQP3 and AQP7 but not AQP9 expression in mature bovine oocytes. In particular, we observed that exposure of oocytes to hyperosmotic Me_2_SO solution could upregulate AQP3 while AQP7 expression was upregulated after EG hyperosmotic exposure. The aquaglyceroporin expression in oocytes after exposure to CPA has previously been reported. Tan et al. [22] demonstrated that murine oocytes exposed to a hyperosmotic CPA solution containing EG, Me_2_SO or sucrose increased the expression of AQP7, but not of AQP3 and AQP9. The immunofluorescence analysis carried out in this study revealed higher levels of AQP3 expression in bovine oocytes exposed to EG than those reported by Tan et al. [38] for mouse oocytes while bovine oocytes exposed to Me_2_SO or EG showed lower AQP7 expression when compared to mouse oocytes. These results suggest that aquaporin upregulation in oocytes due to hyperosmosis may be not only species-specific, but also dependent on the CPA itself.

Upregulation of AQP3 expression in oocytes induced by Me_2_SO is not only due to hyperosmolarity, because upregulation of AQP3 by Me_2_SO was much greater than that induced by sucrose or by EG. The same applies to the upregulation of AQP7 expression by EG. These findings imply that Me_2_SO may boost AQP3 expression or EG may promote AQP7 expression in oocytes not only by increasing osmolarity, but also by directly affecting AQP3 or AQP7 gene transcription and/or translation [38]. The mechanisms involved in CPA-induced AQP expression are unclear. Although previously published studies imply that some transcriptional activity may still occur during this stage [39], the fully developed oocyte is essentially transcriptionally inactive. To precisely regulate the maturation process, oocytes rely on regulation of pre-existing transcripts. As a result, oocyte developmental competence is dependent on both the formation of an adequate pool of RNA in immature oocytes and the highly orchestrated regulation of the stability and processing of these mRNAs during maturation before transcription resumes during embryonic genome activation [40]. The mitogen-activated protein kinase (MAPK) signaling pathway has been found to regulate the stability of mRNA [41]. Hyperosmotic treatment activates the MAPK14/11 pathway in the embryo and in other cell types [42,43]. Moreover, MAPK signaling regulates aquaporin 3 and 9 expression and localization in preimplantation embryo regulation [44]. It is not clear whether the mechanisms involved in the upregulation of aquaporins by CPAs in MII bovine oocytes are similar to those in preimplantation embryos. Tan et al. [22] demonstrated that AQP7 in mouse oocytes may mediate tolerance to hyperosmotic stress during cryopreservation through the activation of the Aurora A/CPEB pathway mediated by PI3K and PKC to upregulate AQP7 expression. Moreover, F-actin may play an important role in AQP7 intracellular trafficking from the ooplasm to the oolemma [22].

Although there have been various studies on the expression and function of AQPs in oocytes, the majority of these studies were focused on AQP3. AQP3 has been found to play a significant role in facilitating the diffusion of water, glycerol and EG but not Me_2_SO or propylene glycol in mouse oocytes [16]. When measured in bovine oocytes, bovine AQP3 has been described to transport water in the presence of glycerol and Me_2_SO but not EG or propylene glycol as occurred in mouse AQP3 [16]. In this study, we examined the permeability of the bovine oocyte in which AQP7 protein was overexpressed. The AQP7 channel was successfully over-expressed in bovine MII oocytes by cRNA microinjection at the germinal vesicle stage. Thus, as with murine, amphibian, and porcine oocytes, bovine oocytes are able to translate exogenous aquaporins during IVM. [16,17,19,45,46].

One limitation of the current study was that differences in AQP7 expression between non-injected and AQP7 injected oocytes were lower than when bovine AQP3 was overexpressed in mouse oocytes [16] or human and zebrafish AQP3 channels were overexpressed in porcine oocytes [17]. This can be explained, at least in part, by the baseline levels of AQP7 expression in bovine oocytes. While AQP expression was zero in mouse or porcine oocytes, bovine oocytes already had native cellular expression of AQP7. Another limitation is that viability or competence of oocytes to resume meiosis in vitro was lower for microinjected oocytes compared to non-injected oocytes, probably caused by the microinjection injury or to the presence of an exogenous cRNA. However, the rate of EGFP protein expression in the injected bovine oocytes was high (≈86%) when compared to previous studies in which AQP3 was microinjected in immature porcine oocytes [17,35], suggesting a higher capacity of bovine oocytes to translate and integrate exogenous aquaporins. Moreover, EGFP is a good proxy for the expression of other proteins, because all of the EGFP-positive oocytes also contained high levels of AQP7 protein located in the ooplasm and around the oolemma.

*L*_p_ values observed in this study for bovine oocytes in a sucrose, EG or Me_2_SO solution were higher than those reported previously [16,47]. Values of *P_s_* for EG and Me_2_SO were also higher than those reported previously by Agca et al. [47] and by Jin et al. [16]. These differences could be due to the use of different mathematical models (the Kedem and Katchalsky (1958) formulation [47]) or to small differences in constants and parameters used to calculate the permeability parameters [16].

Overexpression of AQP7 in bovine oocytes increased water permeability almost two times compared to non-injected oocytes after exposure to Me_2_SO solution, while water permeability remained unchanged after exposure to sucrose and EG solution. This indicates that AQP7 enhances water transport in the presence of Me_2_SO, but not in the presence of EG or sucrose. The results also showed that P_Me2SO_ and P_EG_ values did not differ between non-injected and AQP7 injected oocytes, showing that this aquaporin is not involved in the movement of Me_2_SO and EG. The absence of differences in CPA permeability mediated by the artificial expression of AQP7 could be related to a decreased translation efficiency of AQP7 when expressed in bovine oocytes and/or the limited solute permeability of AQP7. When other channels were studied, different effects on water and CPA permeability were observed. Edashige’s team observed that overexpression of bovine AQP3 in mouse oocytes caused the water permeability to increase in a hypertonic sucrose solution, a glycerol solution, and a Me_2_SO solution, but not in an ethylene glycol solution and a propylene glycol solution [16]. However, *P*_EG_ was 13 times higher in AQP3 overexpressed oocytes. Similarly, porcine oocytes expressing the zebrafish AQP3b-T85A gene and exposed to EG solution allowed water and cryoprotectant to permeate more efficiently [17].

## 5. Conclusions

During cryopreservation, the plasma membrane’s permeability to water and CPAs is critical. However, little is known about the composition of the plasma membrane of bovine oocytes and how its properties affect the oocytes’ water and solute permeability. Membrane channels, like as AQPs, are vital in the cryopreservation of gametes and embryos because they transfer water and non-charged solutes. In the present study, we explored the process by which water and different CPAs move through the oolemma of bovine oocytes, focusing on the role of AQP7 in this movement. First, we observed that in vitro matured oocytes increase expression of AQP3 and AQP7 but not AQP9 when exposed to hyperosmotic solution. Exposure to Me_2_SO upregulates AQP3 expression, while exposure to EG upregulates AQP7 expression in oocytes in a CPA-dependent fashion. Then, we demonstrated that exogenous expression of aquaglyceroporins such as AQP7 is possible in in vitro matured oocytes. When permeability values for membrane transport were assessed, overexpression of AQP7 in bovine oocytes enhanced water permeability about twofold compared to non-injected oocytes after exposure to Me_2_SO solution, while water permeability remained unaltered after exposure to sucrose and EG solution. Therefore, increasing the water permeability of bovine oocytes by overexpressing AQP7 channels could be a good strategy to shorten the time it takes for oocytes to reach osmotic balance, as well as the amount of time they are exposed to the CPA solutions. More research is being conducted to investigate if expression of AQP7 enhances the survival rates of in vitro matured oocytes after cryopreservation. Additionally, knowledge of the biophysical parameters as well as the understanding of the role and modulation of AQP7 in oocyte’s plasma membrane will prompt the development of novel and more effective protocols for bovine oocytes.

## Figures and Tables

**Table 1:** Constant and parameters used in 2P model.

Description	Values	Symbol
Universal gas constant	8.314 m^3^ Pa K^−1^ mol^−1^	*R*
Absolute temperature	298 K	*T*
Partial molar volume of water	18.02 × 10^12^ μm^3^ mol^−1^	*υ_w_*
Partial molar volume of CPA:		*υ_s_*
- EG ^a^ - Me_2_SO ^a^	55.8 × 10^−6^ m^3^ mol^−1^ 71.3 × 10^−6^ m^3^ mol^−1^	
Osmotically inactive volume (MII oocytes):	0.25 [16,32]	*V_b_*

^a^ Partial molar volumes of cryoprotectants from Vian et al. [33]. Abbreviations: CPA, cryoprotectant; EG, ethylene glycol; Me_2_SO, dimethyl sulfoxide; MII, metaphase II.

**Table 2:** Effect of EGFP cRNA injection alone or together with AQP7 cRNA on the survival rate, protein expression and maturation rate at 24 of IVM.

Treatment	*n*	% Survival	% EGFP *	%AQP7 ^§^	% MII ^#^
Non-injected	105	100 ^a^	-	-	74.73 ± 1.93 ^a^
EGFP	84	71.48 ± 8.15 ^b^	88.31 ± 9.36	100	56.55 ± 3.92 ^b^
EGFP+AQP7	135	62.11 ± 2.25 ^b^	82.88 ± 4.25	100	56.54 ± 1.24 ^b^

Unless indicated otherwise, data are given as the mean ± SEM. Within columns, values with different superscript differ significantly (*p* < 0.05). * Rates of oocytes with positive EGFP expression were calculated from the total number of lived oocytes after microinjection. ^§^ Percentage of oocytes expressing AQP7, assessed by immunofluorescence microscopy, with respect to the oocytes expressing EGFP. ^#^ Maturation rate was calculated by using the ratio of oocytes attaining Metaphase II over the total number of lived oocytes.

**Table 3:** Hydraulic conductivity (*L*_p_) of bovine oocytes injected with EGFP alone or EGFP + AQP7 cRNA exposed to HM medium containing 0.5 M sucrose at 25 °C.

Treatment	Sucrose
Non-injected	2.19 ± 0.15 ^a^
EGFP	2.05 ± 0.18 ^a^
EGFP+AQP7	2.17 ± 0.25 ^a^

The *L*_p_ (μm/atm×min) values were assessed from data shown in Figure 4. Data are given as the mean ± SEM. Within columns, values with different superscript letters differ significantly (*p* < 0.05). Non-injected oocytes: *n* = 10; EGFP cRNA-injected oocytes: *n* = 12; and EGFP+AQP7 cRNA-injected oocytes: *n* = 12.

**Table 4:** Hydraulic conductivity (*L*_p_) of bovine oocytes injected with EGFP alone or EGFP + AQP7 cRNA exposed to HM medium containing 9.5% Me_2_SO or 8% EG at 25 °C.

Treatment	Me_2_SO	EG
Non-injected	2.06 ± 0.13 ^a^	1.38 ± 0.12 ^a^
EGFP	1.98 ± 0.24 ^a^	1.44 ± 0.10 ^a^
EGFP+AQP7	3.96 ± 0.57 ^b^	1.39 ± 0.11 ^a^

The *L*_p_ (μm/atm × min) values were assessed from data shown in Figure 5. Data are given as the mean ± SEM. Within columns, values with different superscript letters differ significantly (*p* < 0.05). Me_2_SO: Non-injected oocytes: Me_2_SO *n* = 13; EG *n* = 14; EGFP cRNA-injected oocytes: Me_2_SO *n* = 10; EG *n* = 11; and EGFP+AQP7 cRNA-injected oocytes: Me_2_SO *n* = 11; EG *n* = 13.

**Table 5:** Permeability to cryoprotectants (*P*_s_) of bovine oocytes injected with EGFP alone or EGFP+AQP7 cRNA measured in HM medium containing 9.5% Me_2_SO or 8% EG at 25 °C.

Treatment	Me_2_SO	EG
Non-injected	0.58 ± 0.05	0.78 ± 0.09
EGFP	0.58 ± 0.05	1.04 ± 0.17
EGFP+AQP7	0.57 ± 0.04	0.76 ± 0.10

The *P*_s_ (μm/s) values were determined from the data shown in Figure 5. Data are given as the mean ± SEM. Me_2_SO: Non-injected oocytes: Me_2_SO *n* = 13; EG *n* = 14; EGFP cRNA-injected oocytes: Me_2_SO *n* = 10; EG *n* = 11; and EGFP+AQP7 cRNA-injected oocytes: Me_2_SO *n* = 11; EG *n* = 13.

## Data Availability

All data generated or analyzed during this study are included in this published article.
